# Study—International Multicentric Minimally Invasive Liver Resection (SIMMILR-5): A Comparison of Open, Conventional Laparoscopic and Tele-Robotic Laparoscopic Liver Resection for Hepatocellular Cancer

**DOI:** 10.3390/cancers18061031

**Published:** 2026-03-23

**Authors:** Andrew A. Gumbs, Roland Croner, David Fuks, Hadrien Tranchart, Zacharias Heger Londono, Joseph Derienne, Albert Chomątowski, Amir Nour Mohammadi, Vincent Grasso, Soufyan el Adel, Gianfranco Donatelli, Karol Rawicz-Pruzynski, Mohammad Abu-Hilal, Ibrahim Dagher

**Affiliations:** 1 Hôpital Antoine Béclère, Assistance Publique-Hôpitaux de Paris, 157 Rue de la Porte de Trivaux, 92140 Clamart, France; hadrien.tranchart@aphp.fr (H.T.); joseph.derienne@aphp.fr (J.D.); 2 Department of General-, Visceral-, Vascular- and Transplantation Surgery, University of Magdeburg, 39120 Magdeburg, Germany; 3 Department of Surgical Oncology, Medical University of Lublin, Radziwiłłowska 13 St., 20-080 Lublin, Poland; 4 Department of Visceral Surgery, CHUV–UNIL, Rue du Bugnon 46, 1005 Lausanne, Switzerland; 5 Department of Electrical and Computer Engineering, University of New Mexico, Albuquerque, NM 87106, USA; 6 Department of Medical Education and Scholarship, Rowan-Virtua School of Osteopathic Medicine, Rowan University, Stratford, NJ 08084, USA; 7 Department of Surgery, Amsterdam UMC, University of Amsterdam, 1105 AZ Amsterdam, The Netherlands; 8 Cancer Center Amsterdam, 1081 HV Amsterdam, The Netherlands; 9 Unité d’Endoscopie Interventionnelle, Hôpital Privé des Peupliers, 75013 Paris, France; 10 Department of Clinical Medicine and Surgery, University of Naples “Federico II”, 80125 Naples, Italy; 11 Department of Surgery, School of Medicine, The University of Jordan, Amman 11942, Jordan; 12 Department of Surgery, University Hospital Southampton NHS Foundation Trust, Southampton SO16 6YD, UK

**Keywords:** conventional laparoscopy, digitally enhanced laparoscopy, 3D laparoscopy, collaborative robotic laparoscopy, tele-robotic laparoscopic surgery, hepatocellular (HCC), primary liver cancer, liver resection, hepatectomy, cobotics, cobotic-assisted surgery

## Abstract

Background: The role of minimally invasive surgery (MIS) for hepatocellular carcinoma (HCC) remains controversial because of concerns regarding oncologic adequacy, particularly margin status. While robotic-assisted hepatectomy has gained adoption, its true perioperative advantages over conventional laparoscopy and open surgery remain unclear. SIMMILR-5 was designed to evaluate the short-term outcomes of open, laparoscopic, and tele-robotic laparoscopic hepatectomy for HCC using rigorous adjustment for confounding. Methods: A retrospective international multicenter study was conducted including patients undergoing liver resection for HCC between June 2004 and November 2024 at five high-volume hepatobiliary centers. Surgical approaches included open (O), conventional laparoscopy (L), and tele-robotic laparoscopy (TRL). Propensity score matching was performed using demographic, clinical, and tumor-related variables. The primary endpoint was short-term mortality (30 and 90 days). Secondary outcomes included estimated blood loss (EBL), operative time, length of stay (LOS), R0 resection status, and major complications. Results: A total of 904 patients were identified (302 O, 568 L, 34 TRL). After matching, conventional laparoscopy was associated with significantly lower EBL, shorter operative time, and shorter LOS compared with open surgery (all *p* < 0.00001). Compared with open surgery, TRL was associated with lower EBL but no improvement in operative time or LOS. Compared with laparoscopy, TRL was associated with longer operative time and longer LOS. Short-term oncologic surrogates were comparable across approaches. Conclusions: Minimally invasive hepatectomy offers perioperative advantages over open surgery for selected patients with HCC, driven primarily by conventional laparoscopy. Tele-robotic hepatectomy is feasible and safe in experienced centers but does not demonstrate superiority over advanced laparoscopic techniques.

## Simple Summary

Surgery to remove liver tumors is an important treatment for hepatocellular cancer, but there is ongoing debate about which surgical technique provides the best outcomes. Traditional open surgery has long been considered the standard approach, while minimally invasive methods such as laparoscopy and robotic-assisted surgery have become increasingly common. However, it is still unclear whether these newer techniques provide meaningful advantages for patients. This international multicenter study compared open surgery, conventional laparoscopic surgery, and tele-robotic laparoscopic surgery in patients undergoing liver resection for hepatocellular cancer. By analyzing data from several high-volume surgical centers and adjusting for differences between patient groups, the study aimed to determine whether minimally invasive techniques improve short-term surgical outcomes. The findings suggest that minimally invasive surgery, particularly conventional laparoscopy, can reduce blood loss, shorten surgery time, and decrease hospital stay compared with open surgery, while robotic approaches appear safe but do not currently provide clear additional benefits.

## 1. Introduction

The SIMMILR (Study—International Multicentric Minimally Invasive Liver Resection) was begun to better understand the true advantages, if any, of the complete robotic surgical system [1,2,3]. During SIMMILR-4 it became more obvious that although surgeons were reporting laparoscopic liver resections, many of them were also using aspects of robotics, specifically three-dimensional imaging and/ or a robotic arm to hold the laparoscope [4]. SIMMILR-5 was devised to see if the findings in SIMMILR-4 could be applied to hepatocellular cancer (HCC).

For HCC, the main oncological endpoints include R0 resection rate, preservation of the liver function, and long-term outcomes such as overall survival (OS) and disease-free survival (DFS) [5,6,7]. When R0 resections are achieved, open, laparoscopic and robotic-assisted hepatectomy for HCC have similar outcomes [8,9,10,11]. In general, these are best achieved at high-volume hepatobiliary centers. Notably, there still are no randomized controlled trials demonstrating superior OS or DFS after robotic-assisted liver resections for HCC [12,13].

However, when compared to open surgery, some authors have described decreased estimated blood loss (EBL), transfusion rates, postoperative pain and length of stay (LOS) after robotic-assisted hepatectomy [14,15]. When compared to laparoscopy, robotic assistance may offer improved precision for lesions in the deep/posterior segments. This is believed to be due to the improved dexterity with articulating instruments and better ability to suture minimally invasively [16]. It is important to remember that these potential advantages are only preoperative and technical and not oncologic [17].

Aside from achieving an R0 resection, the fundamental challenges in surgical management of hepatocellular carcinoma relate to precise parenchymal transection, vascular control, and preservation of functional liver reserve [18,19]. Open surgery remains the reference standard for complex resections, particularly for large tumors, lesions in difficult locations, or cases requiring vascular or biliary reconstruction. Minimally invasive approaches, including digitally enhanced laparoscopy (e.g., three-dimensional imaging and indocyanine green fluorescence) and robotic assistance with articulating instruments, may facilitate precise dissection and parenchymal transection compared with standard laparoscopy [20]. However, universally accepted limitations of robotic-assisted hepatectomy for HCC persist, including large tumors (>8 cm), the need for complex vascular or biliary reconstruction, and the presence of advanced multifocal disease [21,22,23]. Because of this, open resection remains the gold standard for these types of cases.

Because most of the literature does not compare open, laparoscopic and robotic-assisted liver resections for hepatocellular cancer, we performed a retrospective, international, multicentric study with propensity score matching for patients undergoing open and minimally invasive liver resection to see if we could better ascertain what benefits, if any, actually exist for robotic-assisted hepatectomy for HCC.

## 2. Methods

### 2.1. Patient Selection and Indication for Surgery

We collected perioperative data as well as follow-up data for all eligible patients who underwent open or MIS for resection of hepatocellular cancer between June 2004 and November 2024. All hepatectomies for hepatocellular cancer were done by surgeons with experience in both open and minimally invasive liver resection at 5 different international centers (2 in France, 1 in Switzerland, 1 in Germany and 1 in Jordan). Minimum case requirements for participating HPB surgeons in this study were completion of at least 50 laparoscopic and/or robotic hepatectomies and at least 50 hepatectomies for cancer.

Surgery was indicated for resectable primary liver cancer, specifically hepatocellular cancer (HCC). Histological confirmation was obtained before surgery when needed. Otherwise, clear imaging evidence of liver cancer was sufficient. However, to ensure patient-centered care, treatment options—perioperative, surgical, or alternative—were reviewed by a multidisciplinary tumor board (MDT) beforehand. Severe lung disease was considered a relative contraindication. Patients were divided into 3 approaches: open (O), conventional laparoscopy (L) and tele-robotic laparoscopic (TRL) resections. All centers did open and L resections and 1 center did TRL. In this study, tele-robotic laparoscopic surgery (TRL) corresponds to what is commonly referred to as robotic-assisted surgery using current telemanipulated platforms (e.g., da Vinci systems), while acknowledging that “robotic-assisted surgery” remains the conventional terminology.

### 2.2. Surgical Definitions

Resections were classified according to the Couinaud liver segmentation system. Major hepatectomy was defined as resection of three or more liver segments. Post-hepatectomy liver failure and bile leak were defined according to the International Study Group of Liver Surgery (ISGLS) criteria. Resection margins were classified as R0 or R1 based on histopathologic assessment, and margin distance was recorded when available. Tumor location was categorized according to segmental anatomy. Deep liver segments were defined as the posterosuperior segments (Couinaud segments VII, VIII, IVa, and I). Tumor location and segment classification were coded using operative reports and imaging review. Conversion to open surgery was defined as any unplanned conversion from minimally invasive to open resection.

### 2.3. Study Endpoints

A retrospective analysis was conducted on patients who received liver resection for hepatocellular cancer. Individuals who had prior Associating Liver Partition and Portal Vein Ligation for Staged Hepatectomy (ALLPS), ablation therapy, or repeat resections were excluded. Cases initiated with a minimally invasive technique were assessed on an intention-to-treat basis. Study data can be accessed from the corresponding author upon reasonable request.

Demographics and confounding variables used for the propensity score matching (PSM) included: age, sex, American Society of Anesthesia (ASA) class, BMI (Body Mass Index) calculated as kg/m^2^, history of previous surgery, neoadjuvant therapy (e.g., chemotherapy, chemoradiotherapy and/or immunotherapy), largest tumor size in mm, number of tumors, location in deep segments and type of liver resection (Major or Minor). The main outcome measured was short-term mortality (death within 30 or 90 days post-surgery). Secondary measures included operative factors (blood loss, OR duration), hospitalization period, R0 resection status, and major complications. The Dindo–Clavien system classified postoperative issues, with severe cases defined as grade ≥ 3. PSM ensured better technique comparison.

The dataset contained no information that could enable patient identification. The STROBE checklist guided manuscript preparation for observational study reporting. Surgical techniques and definition of extent of liver resection have been previously described in SIMMILR 1–4 [1,2,3,4].

### 2.4. Data Analysis

Statistical analyses were performed using Python (version 3.x) and SPSS version 28.0 (IBM Corp., Armonk, NY, USA). Propensity score estimation, matching, and balance diagnostics were conducted in Python, while descriptive and supplementary analyses were performed using SPSS.

Continuous variables were expressed as means ± standard deviation (SD) and categorical variables as frequencies and percentages. For unmatched comparisons, continuous variables were compared using Student’s t-test or Mann–Whitney U test, as appropriate, and categorical variables were analyzed using the chi-square test or Fisher exact test.

Following propensity score matching, statistical comparisons were performed using methods appropriate for matched data. Paired continuous variables were analyzed using paired t-tests or Wilcoxon signed-rank tests, as appropriate. Matched categorical variables were compared using McNemar tests or conditional logistic regression where applicable.

Effect sizes were reported as mean differences for continuous variables and odds ratios for categorical variables, with corresponding 95% confidence intervals. All statistical tests were two-tailed, and a *p*-value < 0.05 was considered statistically significant.

### 2.5. Propensity Score Matching (PSM)

PSM was performed to reduce confounding by indication in this retrospective observational study. PSM is a robust statistical method commonly used in non-randomized settings to emulate some of the balance achieved in randomized controlled trials. In this analysis, the propensity score was estimated using a logistic regression model, where treatment assignment served as the dependent variable and relevant covariates, including demographic, clinical, tumor-related, and procedural characteristics, were included as independent variables. Year of surgery was included as a continuous covariate to account for temporal and learning-curve effects. The goal was to derive a summary score reflecting each patient’s probability of receiving the treatment, conditional on the observed covariates.

Three independent pairwise propensity score matching procedures were performed: (1) open versus conventional laparoscopy, (2) open versus tele-robotic laparoscopy, and (3) conventional laparoscopy versus tele-robotic laparoscopy. For each comparison, a separate logistic regression model was constructed using the same covariate set to ensure methodological consistency across analyses. Matching was performed independently for each comparison; therefore, patients could contribute to more than one matched cohort but were included only once within any individual matched analysis.

After estimating the propensity scores, 1:1 nearest-neighbor matching without replacement was performed to create matched pairs. A caliper width of 0.2 times the standard deviation of the logit of the propensity score was applied to ensure close matches and limit residual imbalance. Patients in the treatment group were matched to those in the control group with similar propensity scores, and unmatched subjects were excluded from the final analysis. Propensity score matching was performed using patient and operative characteristics; however, center-level stratification was not feasible because tele-robotic laparoscopic cases were concentrated in a single center, making center adjustment unstable. Standardized mean differences (SMDs) were calculated before and after matching to assess covariate balance; an SMD less than 0.1 was considered indicative of adequate balance.

Matching diagnostics and visualization techniques, including density plots and Love plots, were used to further validate the adequacy of the matching procedure. Post-matching comparisons of outcomes between groups were conducted using appropriate statistical tests, accounting for the matched design. All propensity score estimation, matching procedures, balance diagnostics, and post-matching analyses were performed using Python (version 3.x) with the pymatch and statsmodels libraries.

This methodological approach was selected to minimize bias from confounding variables and strengthen causal inference regarding the association between the surgical intervention and outcomes. By ensuring that matched groups were well balanced across baseline characteristics, the risk of spurious associations due to selection bias was substantially reduced.

## 3. Results

A total of 904 patients undergoing liver resection for HCC were identified from the five centers: 302 underwent open resection, 568 conventional laparoscopic (L) resection and 34 RAS resection with the four-arm tele-robotic laparoscopic system (TRL). Unlike SIMMILR-4, there were not enough patients undergoing laparoscopy with three-dimensional imaging or collaborative robotic laparoscopy for HCC to allow meaningful analysis of these approaches. After PSM, 208 were in the O vs. L, 32 in the O vs. TRL and 34 in the L vs. TRL groups, respectively. Table 1, Table 2 and Table 3 found no statistically significant differences after PSM in the preoperative demographics and confounding variables; however, as seen below, some variables approached statistical significance.

Table 1, Table 2 and Table 3 show the demographics, confounding and short-term outcome variables for liver resection for hepatocellular cancer (HCC) after propensity score matching, SIMMILR-5 (Study—International Multicentric Minimally Invasive Liver Resection). In Table 1 and Table 3, no results even approached statistical significance. In Table 2, resected lesions tended to be in the deep segments more often in the TRL group when compared to the O group, 31.32% vs. 9.4% (*p* = 0.06), respectively.

Table 4, Table 5 and Table 6 show the short-term outcome variables. All centers in this study that performed TRL used the da Vinci robot (da Vinci, Intuitive Surgical, Sunnyvale, CA, USA).

When the short-term outcome variables were studied, multiple significant and highly significant findings were identified; however, due to the low numbers of patients in the L and TRL groups, findings in Table 5 and Table 6 are mainly considered exploratory (Table 4, Table 5 and Table 6). EBL, LOS and operative times were all highly statistically significantly decreased in the L group when compared to the O group (Table 4). Specifically, EBL was 145 vs. 514 mL, operative time was 302 vs. 347 min and LOS was 8 vs. 10 days, respectively (all *p* < 0.00001).

When O resection was compared with TRL (Table 5), only EBL was significantly lower in the TRL vs. O group, 430 vs. 538 mL, respectively (*p* = 0.01). Although LOS tended to be longer in the TRL group, 15 vs. 10 days, this difference was not statistically significant (*p* = 0.06). Notably, when L was compared to TRL, operative time was significantly longer in the TRL group, 334 vs. 260 min, respectively (*p* = 0.002). In addition, LOS was also longer at 13 vs. 9 days in TRL when compared to L, respectively (*p* = 0.003).

Thirty- and ninety-day mortality outcomes are summarized in Table 4, Table 5 and Table 6. In the open versus laparoscopic comparison, 30-day mortality occurred in 2/208 (1.0%) versus 9/208 (4.4%) patients, and 90-day mortality occurred in 3/208 (1.4%) versus 10/208 (4.8%), respectively. In the open versus tele-robotic laparoscopic comparison, 30-day mortality was 0/32 versus 1/32 (3.1%) and 90-day mortality was 0/32 versus 3/32 (9.4%). In the laparoscopic versus tele-robotic laparoscopic comparison, 30-day and 90-day mortality occurred in 2/35 (5.8%) versus 1/35 (3.0%), respectively. No statistically significant differences in mortality were observed between approaches.

## 4. Discussion

This international multicentric propensity score-matched trial aimed to reveal whether minimally invasive approaches, including conventional (L) and tele-robotic laparoscopy (TRL), have any measurable short-term advantages over open hepatectomy (O) for hepatocellular cancer (HCC). In SIMMILR-5, minimally invasive hepatectomy, particularly conventional laparoscopy, was associated with significantly reduced estimated blood loss, shorter operative times and a decreased length of stay when compared to open hepatectomy. These findings are consistent with previously reported studies showing short-term benefits of MIS, even for complex oncological indications, when performed in high-volume hepatobiliary centers [21,24,25,26,27]. Notably, these technical advantages were seen without any apparent compromise in terms of short-term oncological outcomes, thus indicating that the minimally invasive approach is suitable for selected patients with HCC [28].

However, when TRL hepatectomy was analyzed separately, the expected benefits over conventional laparoscopy were not observed. Although TRL was associated with a reduced EBL when compared to open surgery, no significant advantages were noted in the operative time or the LOS. In fact, there was a tendency for LOS to be longer when compared to open hepatectomy. When these operative time and LOS after TRL were compared to conventional laparoscopy, both were significantly longer after TRL. These observations suggest that the perceived technical advantages of current TRL platforms, such as improved dexterity and enhanced visualization, may not consistently translate into superior short-term outcomes for patients with HCC when compared to laparoscopy [29,30,31].

The longer length of stay observed in the tele-robotic laparoscopic group should be interpreted cautiously. This finding may reflect institutional perioperative pathways, patient selection, postoperative monitoring protocols, or discharge practices rather than the surgical approach itself. In particular, because tele-robotic procedures were predominantly performed at a single center, differences in enhanced recovery protocols or discharge criteria may have influenced LOS. Therefore, LOS differences may represent center-specific practices rather than an inherent limitation of the tele-robotic approach.

These observations highlight broader issues in the published literature surrounding TRL liver resection. Much of the published data comes from studies from single centers or heterogeneous multicenter series without rigorous adjustment for confounding. As demonstrated in this study, propensity score matching in an international multicentric trial may attenuate many of the reported advantages of TRL hepatectomy. These findings suggest that some of these previously reported benefits may, in fact, reflect selection or reporting bias and not platform-specific benefits [1,2,3,4].

Although there may be short-term benefits in selected patients, oncological outcomes after TRL appear comparable. Furthermore, there is a strong dependence on institutional and surgeon experience for these benefits to be realized with TRL. In this study, minimally invasive hepatectomy for HCC was associated with decreased EBL. Notably, no benefits were seen after TRL compared to open hepatectomy after this multicentric international PSM. Furthermore, patients undergoing conventional laparoscopy (L) also had improved operative times and shorter LOS when compared to TRL. These findings highlight the potential bias in reporting, particularly in single institution reports, in non-international multicentric reports that do not perform PSM and in studies that do not compare laparoscopy with TRL [32,33,34,35,36]. Most studies consistently demonstrate the feasibility and safety of TRL hepatectomy for HCC; however, it is still unclear if the platform itself offers any true benefit to this patient population when compared to laparoscopy [1,2,3,4].

Although mortality differences were not statistically significant, the numerically higher 30-day and 90-day mortality observed in the laparoscopic group compared with the open group warrants cautious interpretation. Given the small number of events and the limited power to detect differences in rare outcomes, these findings likely reflect random variation rather than true inferiority of laparoscopy. Residual confounding, including differences in underlying liver function not fully captured in the dataset, cannot be excluded.

It is also important to contextualize these results within the evolving definitions of minimally invasive, robotic-assisted, and collaborative robotic surgery. As discussed in recent consensus efforts, including those of the Artificial Intelligence Organization of the Next Generation of Surgeons (AIONS), current tele-robotic systems function primarily as advanced tele-manipulators with limited autonomy [37]. Consequently, distinctions between conventional laparoscopy, digitally enhanced laparoscopy, and tele-robotic approaches may be less pronounced in clinical outcomes than often assumed [4]. Future generations of robotic systems incorporating higher levels of autonomy or true collaborative interaction may yield different results.

### 4.1. Limitations

This study has several important limitations. The retrospective and observational design inherently introduces the risk of selection bias and unmeasured confounding, despite the use of rigorous propensity score matching (PSM) to balance baseline demographic, clinical, and tumor-related variables. Although PSM improves comparability between groups, it cannot fully replicate the balance achieved by prospective randomized controlled trials [38]. Certain perioperative variables, including Pringle maneuver use, intraoperative transfusion requirements, and some detailed liver-specific outcomes, were not consistently available across all centers and therefore could not be analyzed. The use of hepatectomy complexity scores like the IWATE scoring system may also enable researchers to do fairer comparisons and obtain more accurate results [39].

The tele-robotic laparoscopic (TRL) cohort was relatively small compared to the open (O) and conventional laparoscopic (L) groups, reflecting the limited global adoption of robotic hepatectomy for hepatocellular cancer (HCC). Furthermore, the tele-robotic laparoscopic cohort was derived predominantly from a single center and included a limited number of cases. Although adjustment for the year of surgery was performed to account for temporal effects, institutional practice patterns and perioperative pathways may still influence outcomes and introduce residual center-level confounding not fully addressed by propensity score matching. As a result, analyses involving TRL, particularly comparisons with conventional laparoscopy, should be interpreted as exploratory and hypothesis-generating rather than definitive. The limited sample size may also reduce statistical power to detect differences in uncommon but clinically meaningful outcomes such as major morbidity and mortality.

This was an international multicenter study involving high-volume hepatobiliary centers with significant experience in both open and minimally invasive hepatectomy. While this enhances internal validity and technical consistency, it may limit generalizability to lower-volume centers or institutions earlier in their learning curve for minimally invasive liver surgery. Surgeon expertise and institutional experience remain critical determinants of outcomes, particularly for complex procedures such as hepatectomy for HCC.

Heterogeneity in perioperative management, including the implementation of enhanced recovery after surgery (ERAS) protocols, postoperative care pathways, and discharge practices across participating centers, may have influenced secondary outcomes such as length of stay. Although these variations reflect real-world practice, they may confound the interpretation of differences unrelated to surgical approach alone.

Finally, the present analysis focused exclusively on short-term perioperative outcomes. Long-term oncologic endpoints, including disease-free survival and overall survival, were not evaluated [40]. Consequently, conclusions regarding oncologic equivalence between surgical approaches should be interpreted in the context of the existing literature rather than inferred directly from these data [41].

### 4.2. Future Directions

We previously identified five ways of doing liver resection: open (O), conventional laparoscopy (L), digitally enhanced laparoscopy with three-dimensional imaging (DEL), collaborative robotic laparoscopy with a single robotic arm (CRL) with tele-robotic laparoscopic surgery with 3D imaging and four robotic arms (TRL) [4]. L, DEL, CRL and TRL are all various forms of minimally invasive surgery (MIS). At the end of the day, all four MIS approaches during abdominal surgery are technically forms of laparoscopy. Currently, the definition of robotic and robotic-assisted surgery (RAS) is poorly understood. RAS (a.k.a. TRL) systems use so-called complete robotic surgical systems, which have three-dimensional (3D) imaging, a robotic arm holding the 3D laparoscope and 2–4 remote-controlled articulating instruments. Unlike TRL, CRL does not use a remote-controlled platform and the surgeon works locally in contact with the patient and the robotic arm.

At a recent Artificial Intelligence Organization of the Next Generation of Surgeons (AIONS) consensus conference on Definitions of Artificial Intelligence Surgery, Surgomics and Robotics held at the botanical garden of the University of Padova, collaborative robotics (cobotics) was differentiated from TRL in several aspects [37]. Participants reasoned that there must be an element of collaboration between the surgeon and robot, specifically that the robot should have level 2–4 surgical autonomy. However, this creates a bit of a conundrum, because, as mentioned, current RAS/TRL/CRSS are principally tele-manipulators with the surgeon (human) controlling all aspects of the robot’s movements. Furthermore, true robotic surgery was defined as level 5 surgical autonomy where no human control is necessary to perform a surgical procedure. To help resolve this, the consensus agreed that the term RAS should be reserved for surgical robots with level 1 surgical autonomy (e.g., tele-manipulation only).

Therefore, what should cobotic-assisted surgery (CAS) mean? The CRL cases in SIMMILR-4 were done with either a foot pedal or voice control controlling the cobotic arm and, thus, the laparoscope. CAS could then be a surgeon local to the arm and patient, but tele-manipulating a laparoscope with either their foot, voice or other manner. Some companies have developed tool-tracking technology where the laparoscope’s movements follow pre-determined instruments via the use of bounded boxes. Although this would, on the surface, seem to be a higher level of surgical autonomy, it is in fact simply another form of remote control.

## 5. Conclusions

Taken together, the present study demonstrates that minimally invasive hepatectomy offers clear perioperative advantages over open surgery for selected patients with hepatocellular cancer. These benefits, however, appear to be driven primarily by conventional laparoscopy rather than by tele-robotic laparoscopic assistance. While TRL hepatectomy for HCC is feasible and safe in experienced, high-volume centers, it does not currently confer demonstrable superiority over advanced laparoscopic approaches with respect to short-term outcomes.

Accordingly, TRL/robotic-assisted hepatectomy for HCC should be viewed as a complementary technique rather than a replacement for conventional laparoscopy. Future advances in robotic technology, particularly those incorporating genuine autonomy and true surgeon–robot collaboration, may alter this balance. Until such developments are realized and supported by high-quality comparative evidence, careful patient selection, surgeon expertise, and institutional experience remain the most important determinants of outcomes in minimally invasive liver surgery for HCC.

## Figures and Tables

**Table 1:** Demographics and confounding variables after propensity score matching, SIMMILR-5.

Variable	Open (O) (n = 208)	Laparoscopic (Lap) (n = 208)	Effect Size (95% CI)	*p*-Value
Age, mean ± SD	67.7 ± 10.7	69.1 ± 10.4	−0.13 (−0.31 to 0.06)	0.6
Male:Female	168:40	176:32	3.8% (−3.8% to 11.6%)	0.4
ASA III–IV (%)	96 (46.2)	88 (42.3)	1.2 (0.8–1.7)	0.8
BMI (kg/m^2^), mean ± SD	27 ± 3.7	27.1 ± 3.8	−0.03 (−0.21 to 0.16)	0.6
Previous Surgery (%)	64 (30.1)	60 (28.8)	−1.3% (−7.6% to 10.2%)	0.7
Neoadjuvant TACE Therapy (%)	25 (12.0)	19 (9.1)	2.9% (−2.2% to 8.0%)	0.4
Metastasis Size (mm), mean ± SD	28.6 ± 37.0	27.4 ± 26.3	0.04 (−0.15 to 0.23)	0.3
Number of Tumors ± SD	1.3 ± 0.9	1.6 ± 2.7	−0.30 (−0.7 to 0.09)	0.5
Deep Segment Location (%)	129 (62)	106 (51)	5.3% (−3.7% to 14.3%)	0.2
Major Resection (%)	89 (43)	98 (47)	−1.9% (−9.9% to 6.1%)	0.6

Open (O) vs. laparoscopic (Lap) [(O) (n = 208), (Lap) (n = 208)]. ASA = American Society of Anesthesia; BMI = Body Mass Index.

**Table 2:** Demographics and confounding variables after propensity score matching, SIMMILR-5.

Variable	Open (O) (n = 32)	TRL (n = 32)	Effect Size (95% CI)	*p*-Value
Age, mean ± SD	64.2 ± 10.5	68.4 ± 7.7	−4.2 (−9.4 to +1.0)	0.1
Male:Female	26:6	26:6	0% difference	1
ASA I–IV (%)	II = 14; III = 18	II = 14; III = 18	No difference	1
BMI (kg/m^2^), mean ± SD	28.9 ± 3.3	28.8 ± 4.2	+0.1 (−1.9 to +2.1)	0.9
Previous Surgery (%)	11 (34.4)	8 (25)	+9.4% (−10.4% to +29.3%)	0.6
Neoadjuvant TACE Therapy (%)	4 (12.5)	3 (9.4)	+3.1% (−11.6% to +17.8%)	1
Metastasis Size (mm), mean ± SD	40.3 ± 42.2	48.1 ± 33.5	−7.8 (−20.8 to +5.2)	0.1
Number of Tumors ± SD	1.6 ± 1.0	1.3 ± 0.8	+21.9% (−0.7% to +44.5%)	0.6
Deep Segment Location (%)	3 (9.4)	10 (31.3)	+21.9% (−0.7% to +44.5%)	0.06
Major Resection (%)	3 (9.4)	10 (31.3)	−4.2 (−9.4 to +1.0)	0.06

Open (O) vs. tele-robotic laparoscopic (TRL) surgery [(O) (n = 32), (TRL) (n = 32)]. ASA = American Society of Anesthesia; BMI = Body Mass Index.

**Table 3:** Demographics and confounding variables after propensity score matching, SIMMILR-5.

Variable	Laparoscopic (Lap) (n = 34)	Tele-Robotic Laparoscopic (TRL) (n = 34)	Effect Size (95% CI)	*p*-Value
Age, mean ± SD	62.6 ± 11.9	67.3 ± 9.1	4.7 (−1.3 to 10.7)	0.1
Male:Female	23:12	28:7	1.8 (0.6–5.3)	0.3
ASA III–IV (%)	18 (52.9)	19 (55.9)	0.9 (0.3–2.3)	0.6
BMI (kg/m^2^), mean ± SD	27.7 ± 4.3	29.0 ± 4.1	1.3 (−0.6 to 3.2)	0.3
Previous Surgery (%)	11 (32.3)	9 (26.5)	0.8 (0.3–2.2)	0.8
Neoadjuvant TACE Therapy (%)	4 (11.8)	3 (8.8)	0.7 (0.4–3.1)	1
Metastasis Size (mm), mean ± SD	50.3 ± 33.1	47.5 ± 29.0	−2.8 (−19.1 to 13.5)	0.9
Number of Tumors ± SD	1.2 ± 1.0	1.5 ± 0.8	0.3 (−0.03 to 0.6)	0.1
Deep Segment Location (%)	10 (29.4)	11 (32.3)	1.1 (0.4–3.1)	0.8
Major Resection (%)	8 (23.5)	10 (29.4)	1.4 (0.5–4.0)	0.8

Laparoscopic (Lap) vs. tele-robotic laparoscopic (TRL) surgery [(Lap) (n = 34), (TRL) (n = 34)]. ASA = American Society of Anesthesia; BMI = Body Mass Index.

**Table 4:** Short-term outcome variables after propensity score matching, SIMMILR-5.

Variable	Open (O) (n = 208)	Laparoscopic (Lap) (n = 208)	Effect Size (95% CI)	*p*-Value
Estimated Blood Loss (mL), mean ± SD	513.8 ± 445.0	369.3 ± 397.2	144.5 (63.5–225.5)	<0.00001
Operative Time (min), mean ± SD	346.6 ± 43.1	302 ± 94.4	44.6 (30.5–58.7)	<0.00001
Length of Stay (days), mean ± SD	9.8 ± 4.4	8.3 ± 8.4	1.5 (0.21–2.79)	<0.00001
Conversion Rate (%)	NA	9 (4.3)	NA	NA
Clavien–Dindo >= Grade III morbidity (%)	22 (10.6)	20 (9.6)	1.11 (0.6–2.1)	0.7
30-day mortality (%)	2 (1.0)	9 (4.4)	0.21 (0.05–1.0)	0.06
90-day mortality (%)	3 (1.4)	10 (4.8)	0.29 (0.08–1.1)	0.09
R1 Resection (%)	25 (12.0)	27 (13.1)	0.92 (0.5–1.6)	0.9

Open (O) vs. laparoscopic (Lap) [(O) (n = 208), (Lap) (n = 208)].

**Table 5:** Short-term outcome variables after propensity score matching, SIMMILR-5.

Variable	Open (O) (n = 32)	Tele-Robotic Laparoscopic (TRL) (n = 32)	Effect Size (95% CI)	*p*-Value
Estimated Blood Loss (mL), mean ± SD	537.5 ± 284.0	430 ± 333.7	107.5 (−44.3–259.3)	0.01
Operative Time (min), mean ± SD	348.6 ± 52.9	326.7 ± 93.0	21.9 (−15.1–58.9)	0.5
Length of Stay (days), mean ± SD	9.6 ± 2.3	14.9 ± 12.0	−5.3 (−9.5–1.1)	0.06
Conversion Rate (%)	NA	5 (15.6)	NA	NA
Clavien–Dindo >= Grade III morbidity (%)	5 (36.3)	8 (25)	0.56 (0.17–1.86)	0.5
30-day mortality (%)	0	1 (3.1)	0.33 (0.01–8.18)	1
90-day mortality (%)	0	3 (9.4)	0.14 (0.01–2.54)	0.2
R1 Resection (%)	2 (6.3)	0	5.10 (0.24–111.0)	0.5

Open (O) vs. tele-robotic laparoscopic (TRL) surgery [(O) (n = 32), (TRL) (n = 32)].

**Table 6:** Short-term outcome variables after propensity score matching, SIMMILR-5.

Variable	Laparoscopic (L) (n = 34)	Tele-Robotic Laparoscopic (TRL) (n = 34)	Effect Size (95% CI)	*p*-Value
Estimated Blood Loss (mL), mean ± SD	527.4 ± 726.1	465.6 ± 345.1	61.8 (−293.6 to 417.2)	0.2
Operative Time (min), mean ± SD	260.1 ± 112.6	334.4 ± 99.4	74.3 (28.2 to 120.4)	0.002
Length of Stay (days), mean ± SD	9.1 ± 10.7	13.0 ± 9.9	3.9 (1.4 to 6.4)	0.003
Conversion Rate (%)	3 (8.8)	3 (8.8)	1.0 (0.20 to 5.0)	1
Clavien–Dindo >= Grade III morbidity (%)	8 (23.5)	6 (17.6)	0.71 (0.22 to 2.28)	0.8
30-day mortality (%)	2 (5.8)	1 (3.0)	0.49 (0.04 to 5.61)	1
90-day mortality (%)	2 (5.8)	1 (3.0)	0.49 (0.04 to 5.61)	1
R1 Resection (%)	3 (8.8)	2 (5.9)	0.65 (0.10 to 4.19)	1

Laparoscopic (L) vs. tele-robotic laparoscopic (TRL) surgery [(L) (n = 34), (TRL) (n = 34)].

## Data Availability

Data is available upon reasonable request.
